# Progress in Research on the Preparation of Graphene-Based Aerogels Using γ-ray Irradiation Technology

**DOI:** 10.3390/gels10020090

**Published:** 2024-01-24

**Authors:** Kai Fan, Lin Lin, Danyi Li, Fangzheng Wang, Jihao Li

**Affiliations:** 1 School of Architecture and Materials, Chongqing College of Electronic Engineering, Chongqing 401331, China; fankai20022002@163.com; 2 Shanghai Institute of Applied Physics, Chinese Academy of Sciences, Shanghai 201800, China; linlin@sinap.ac.cn (L.L.); lidanyi@sinap.ac.cn (D.L.); wangfangzheng@sinap.ac.cn (F.W.)

**Keywords:** graphene aerogels, irradiation, γ-ray, composites

## Abstract

Graphene aerogels (GAs) are of significant interest in the scientific community due to their unique attributes, including a three-dimensional porous structure, exceptional specific surface area, and remarkable chemical stability. Researchers have made notable breakthroughs in aerogel preparation, focusing on aspects like porous structures and chemical stability. This review explores product morphologies and properties developed between 2011 and 2023, particularly examining applications of graphene aerogels with amine or alcohol radical scavengers. It offers a roadmap for researchers, suggesting possibilities for radiation-based preparation and indicating broader applications. These findings contribute to a deeper understanding of aerogels and expand the potential applications of graphene aerogels across various fields.

## 1. Introduction

Graphene aerogel (GA) stands out as an extraordinary three-dimensional porous solid material, distinguished by its ultra-low density, impressive specific surface area, exceptional porosity, and remarkable adsorption capacity. These remarkable properties bestow upon GA a wide spectrum of applications, encompassing energy storage [1,2], catalysis [3,4], sensors [5], environmental protection [6], and beyond. When compared to pristine graphene, GA triumphs over the challenges of agglomeration and the reduction in specific surface area, with issues often stemming from the formidable π-π conjugation and van der Waals forces between graphene sheets during processing. This triumph significantly broadens the horizons for graphene applications, such as energy storage, thermal insulation, and environmental protection. Despite its many potential applications, GA is still expensive to produce and there are no widely available graphene aerogel products on the market today. Therefore, future research and development must focus on reducing production costs, improving efficiency, and developing new application areas.

Traditional synthesis methods for GA encompass hydrothermal reduction, template-based approaches, and sol-gel methods [7]. Among these methods, the hydrothermal reduction method reigns as the most prevalent. It commences with graphene oxide (GO), initiating a process of thermal reduction and self-assembly or chemical cross-linking of GO in aqueous or organic solutions to yield graphene hydrogel (GH) [8]. Subsequently, GA can be meticulously crafted through the freeze-drying or supercritical-CO_2_-drying of GH, thus realizing its full potential in a myriad of applications [9,10].

Gamma-rays, as a form of ionizing radiation, are characterized by their high energy and are primarily emitted during the decay reactions of radionuclides such as ^60^Co and ^137^Cs. When γ-rays interact with various forms of matter, including aqueous solutions, they have the capability to ionize or excite atoms and molecules, resulting in the generation of active particles. This, in turn, initiates a cascade of physical and chemical reactions.

In recent times, the utilization of γ-ray irradiation techniques has gained considerable popularity due to their efficiency, convenience, and ease of use. This technique has found widespread application in the fabrication and modification of inorganic nanomaterials [11,12], as well as the development of polymer functional materials [13,14]. Researchers have harnessed the power of γ-ray irradiation to precisely tailor the properties of these materials for specific applications.

Furthermore, it is worth noting that the versatility of γ-ray irradiation extends to the realm of graphene-based materials. Several studies have demonstrated the successful preparation and enhancement of graphene-based materials using γ-ray irradiation. This innovative approach has shown promise in unlocking new possibilities for graphene-based materials, making them even more versatile and adaptable to a wide range of technological and scientific advancements [15,16,17,18]. However, only a few works have summarized the graphene aerogels prepared using conventional chemical methods and few studies have been reported on the preparation of GAs by γ-ray irradiation.

**Figure 1:**
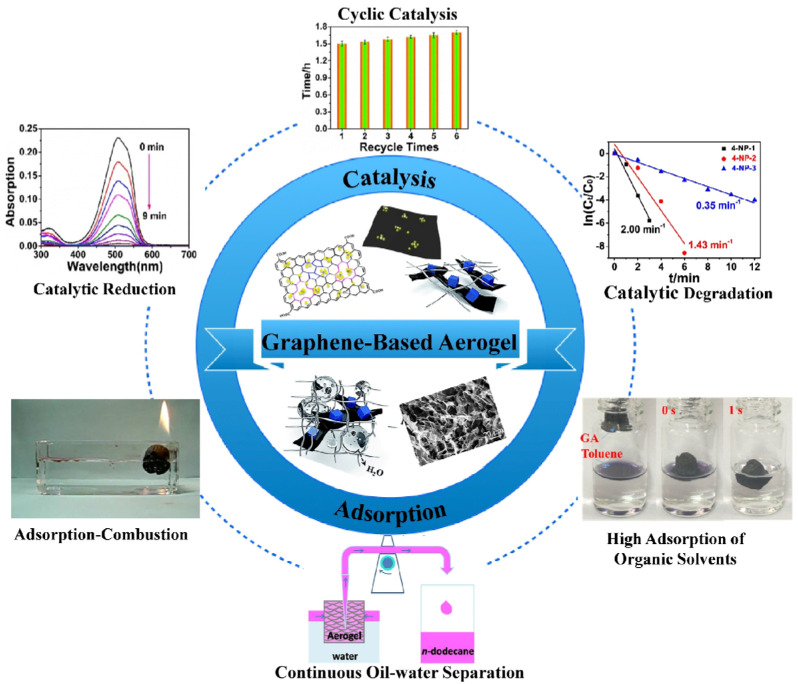
Catalysis and absorption applications of graphene-based aerogels.

Gamma-ray irradiation provides a uniform reduction in GO, ensuring consistent and controlled reduction across the entire graphene structure. This results in a more homogeneous and well-defined GA. The use of γ-ray irradiation helps prevent the agglomeration of reduced graphene oxide sheets. This results in a well-dispersed and interconnected graphene network within the aerogel, contributing to improved mechanical and thermal properties. As depicted in Figure 1, the graphene-based aerogels, meticulously crafted through the powerful technique of γ-ray irradiation, exhibit an exceptional degree of reduction and feature a captivating 3D honeycomb-like structure. This structural marvel serves as a testament to the ingenuity of the fabrication process.

These remarkable graphene-based aerogels, hereinafter referred to as GA, offer a plethora of applications driven by their distinctive characteristics. Of particular note are their remarkable roles in catalysis and adsorption. Within the realm of catalysis, GA demonstrates the capability to facilitate the catalytic reduction in various organic substances, thereby contributing to the advancement of green and sustainable chemical processes. Simultaneously, in the realm of adsorption, GA’s unique properties enable its use in the continuous and efficient separation of oil and water, a crucial process in addressing environmental challenges.

This comprehensive review is designed to equip readers with a profound understanding of the cutting-edge developments in the preparation of GA using γ-ray irradiation across diverse solvent systems. By delving into the latest advancements, readers can gain valuable insights into the intricate interplay between materials science and radiation-based techniques, with the potential to unlock innovative solutions for challenges in catalysis, adsorption, and beyond.

## 2. Radiation Synthesis of GA

**Scheme 1:**
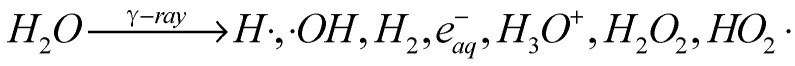
Radiolysis mechanism of water.

Under the influence of γ-ray irradiation, as thoughtfully illustrated in Scheme 1, water undergoes a fascinating transformation, generating a multitude of active products. Among these products are the formation of H_2_ and H_2_O_2_ molecules, the emergence of hydrated electrons (e_aq_^−^), and the release of ·OH, H·, HO_2_· radicals, and H_3_O^+^. This diverse array of species plays a pivotal role in the ensuing chemical and physical reactions.

Notably, within this intricate interplay of reactions, there exists a delicate balance between reductive and oxidative species. Hydrated electrons and H· are representative of the reductive species, while ·OH, HO_2_·, and H_2_O_2_ fall within the category of oxidative species. However, it is within this dynamic equilibrium that the presence of radical scavengers in aqueous systems, such as alcohols or amines, becomes significant. When introduced, these scavengers play a remarkable role in eliminating and converting oxidative radicals, particularly the highly reactive ·OH, into reductive radicals. These reductive radicals synergize with the potent e_aq_^−^ to engage in the reduction of oxidative substances, shaping the complex chemistry of the irradiation process.

**Figure 2:**
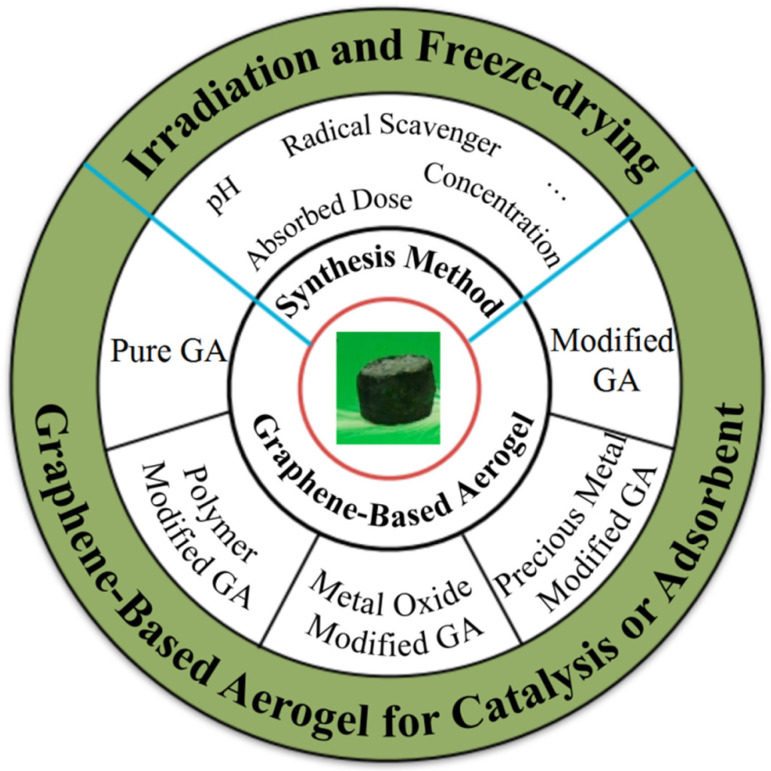
The synthesis method of graphene-based aerogels and their applications.

This intricate dance of chemical reactions has profound implications for the graphene-based aerogels prepared through the dual techniques of γ-ray irradiation and freeze-drying. These materials are subject to various influencing factors, including pH and the type of radical scavengers, as elegantly depicted in Figure 2. The precise manipulation of these factors allows for the fine-tuning of GA’s properties, rendering it a versatile tool in the realms of catalysis and adsorption.

Furthermore, the versatility of the γ-ray irradiation process extends even further. Through the introduction of different modified species during irradiation, GA can be endowed with enhanced catalytic or adsorption properties, thus expanding its application horizons. This multifaceted approach enables the tailoring of graphene-based aerogels to meet the demands of a diverse range of applications, with the potential for groundbreaking contributions to science and technology.

### 2.1. Pure GA

In a recent breakthrough, He et al. [19] introduced a novel and remarkably straightforward approach for the fabrication of graphene-based aerogels using GO dispersions in ethylenediamine (EDA). This method involves a two-step process that ultimately yields reduced graphene oxide (RGO) aerogels characterized by their lightweight nature and high electrical conductivity.

The initial step of the process involves the generation of GH through ^60^Co γ-ray irradiation-induced reduction, accompanied by the deoxygenation process achieved by bubbling nitrogen into the mixture. Subsequently, GH is subjected to freeze-drying, resulting in the formation of RGO aerogels with an impressive array of properties.

Notably, EDA plays a pivotal role in this process by acting as a radical scavenger. EDA captures the ·OH radicals generated during the water radiolysis process, thus eliminating oxygen-containing functional groups from the GO sheets. Concurrently, EDA introduces nitrogen-related groups, effectively transforming the GO sheets. This multifaceted role of EDA in the reduction process leads to the formation of RGO aerogels with tailored properties.

**Figure 3:**
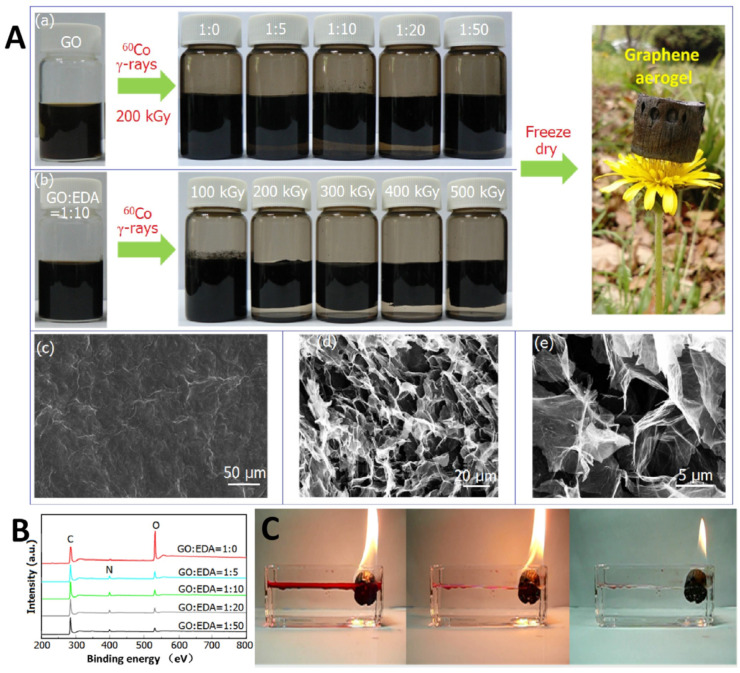
(**A**) Preparation of GA by irradiation reduction in an ethylenediamine environment. (**B**) The C/O ratio in XPS changes with the addition of ethylenediamine. (**C**) Adsorption of organic solvents and stable combustion. (Reproduced from Refs. [19,20] with permission).

As part of their comprehensive exploration, the authors [20] delve deeper into the effects of EDA dosage and γ-ray absorbed dose on the reduction degree of GO and the self-assembly process (Figure 3). Their experimental findings highlight the necessity of a specific EDA content and absorbed dose for the successful formation of RGO hydrogels. The C/O ratio of RGO increases with higher EDA content, indicative of an enhanced reduction degree of GO. Moreover, EDA’s radical scavenging effect influences the self-assembly process of GO sheets, emphasizing the delicate balance required for the formation of RGO.

Interestingly, the increase in the absorbed dose proves advantageous, resulting in various benefits such as the expansion of reducing radical types, the enhancement of GO reduction, the restoration of the lattice structure, and the improvement in thermal stability. At an absorbed dose of 400 kGy, the C/O ratio reaches its peak value of 9.56 (as presented in Table 1), while exhibiting minimal heat loss during heating.

Furthermore, the hydrophobic and lipophilic nature of RGO has led the authors to explore its application in oil–water separation. RGO aerogels demonstrate the ability to quickly adsorb and collect high-density organic liquids, highlighting their potential for environmental applications. Moreover, in adsorption–combustion tests, RGO aerogels exhibit the capability to selectively absorb organic liquids while repelling water, making them suitable for the collection and recycling of spilled oil, further underlining their versatility and environmental relevance.

When alcohol (ROH) is employed as a free radical scavenger, it plays a crucial role in quenching highly reactive ·OH by donating one of its hydroxyl hydrogen atoms, leading to the formation of the corresponding alcohol radical (RO·). The stability of this alcohol radical, such as in the cases of isopropanol and t-butanol, is significantly influenced by the nature of the attached alkyl group. These alkyl groups contribute to the stability of the alcohol radicals through the hyperconjugative effect, thereby enhancing the scavenging efficiency of ·OH radicals.

**Figure 4:**
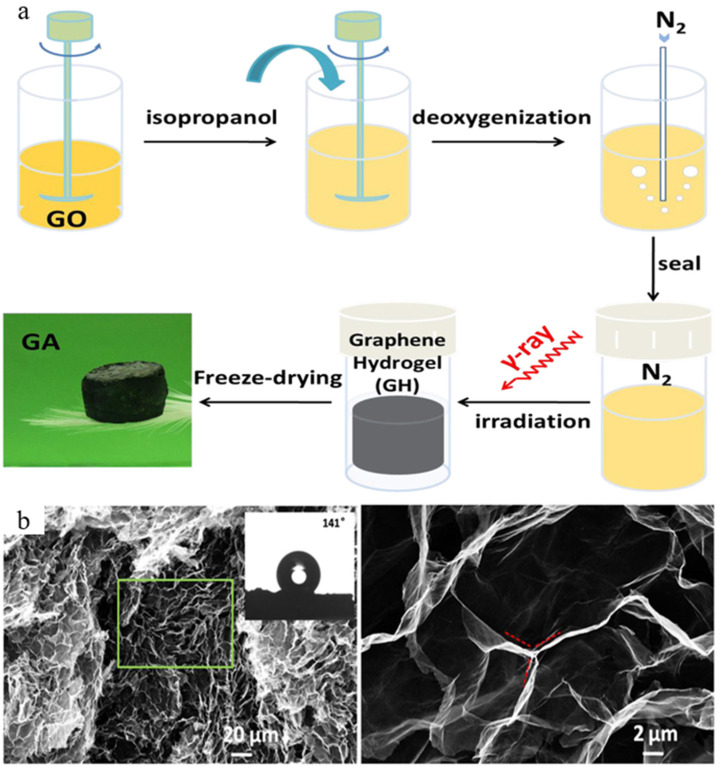
(**a**) Preparation of GA by irradiation reduction in an isopropanol environment. (**b**) GA with high porosity and hydrophobicity. (Reproduced from Ref. [21] with permission).

In a noteworthy study conducted by He et al. [21] in 2016, the scavenging capabilities of isopropanol were harnessed to efficiently reduce GO under ^60^Co γ-ray irradiation (Figure 4). This innovative approach yielded GA with remarkable characteristics, including high porosity and hydrophobicity. During the reduction process, a substantial number of new graphitic domains were introduced into the GA structure, resulting in an increased defect density. Additionally, the carbon-to-oxygen (C/O) ratio was significantly improved, escalating from 2.1 to an impressive 12.0.

Furthermore, it is important to note that the thermal stability of the resulting graphene aerogel was notably enhanced. This improvement can be attributed to the pyrolysis of oxygen-containing functional groups within the material during heating. Despite these advancements, it is worth mentioning that the thermal stability of GA remains somewhat lower when compared to that of pristine graphite. However, the unique properties of GA make it a promising candidate for a wide range of applications in various fields.

**Figure 5:**
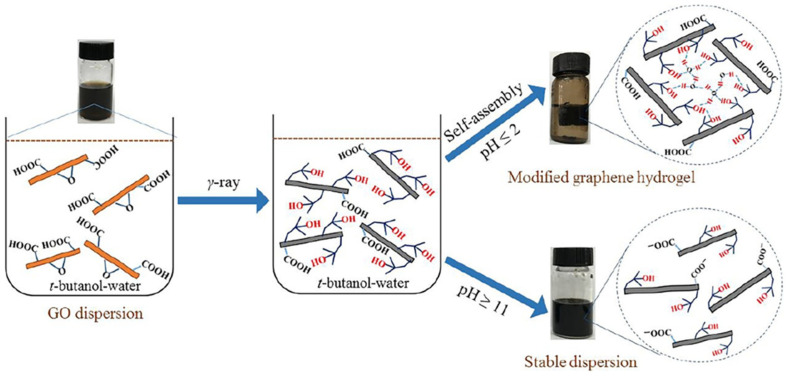
GA exhibits pH-dependent assembly behavior, resulting in completely different product morphologies under strong acidic and alkaline conditions. (Reproduced from Ref. [22] with permission).

The preparation process of GAs is a meticulous endeavor where the choice of alcohols and the pH of the alcohol/water solution play pivotal roles in shaping the reduction and self-assembly outcomes of GO nanosheets. In a significant development in 2017, Wang et al. [22] adopted a similar approach, combining γ-ray irradiation and freeze-drying to fabricate free-standing macroporous graphene aerogels using a t-butanol/water medium. Their study unveiled a fascinating aspect of this process, demonstrating that the hydroxyalkylation degree of GO nanosheets induces pH-dependent assembly behaviors, resulting in strikingly different product morphologies under strongly acidic and basic conditions (Figure 5).

**Scheme 2:**
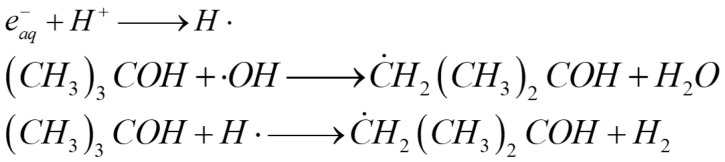
Reactions of primary radiolysis active species in *t*-butanol/water solution.

When the pH of the t-butanol/water solution fell within the range of 2 to 11, the GO nanosheets exhibited the ability to form a uniform dispersion. However, it is important to note that the self-standing graphene aerogel was only obtained in a medium with a pH lower than 2. In strongly acidic conditions, the radiolysis of t-butanol generated a relatively high concentration of ·CH_2_(CH_3_)_2_COH. This occurred because e_aq_^−^ was entirely converted into H· under these conditions. This elevated hydroxyalkylation of GO nanosheets (as depicted in Scheme 2) triggered a remarkable response. Driven by a combination of strong π-π conjugation between the nanosheets and hydrogen bonds formed by the -OH group and water molecules, the nanosheets self-assembled to create a monolithic graphene aerogel structure.

In contrast, under strongly basic conditions, the hydroxyalkylation degree of GO nanosheets was relatively low. The ionization of remaining oxygen-containing groups, such as—COOH, led to electrostatic repulsion, enhancing the stability of the nanosheets. As a result, the nanosheets displayed minimal changes even after irradiation. This exquisite interplay of pH, alcohol choice, and the radiolysis process offers a powerful avenue for tailoring the properties and structures of graphene aerogels to meet specific application requirements, making this an exciting area of research with promising implications in materials science.

**Scheme 3:**
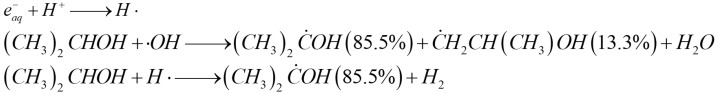
The reaction mechanism of isopropanol as an example.

**Figure 6:**
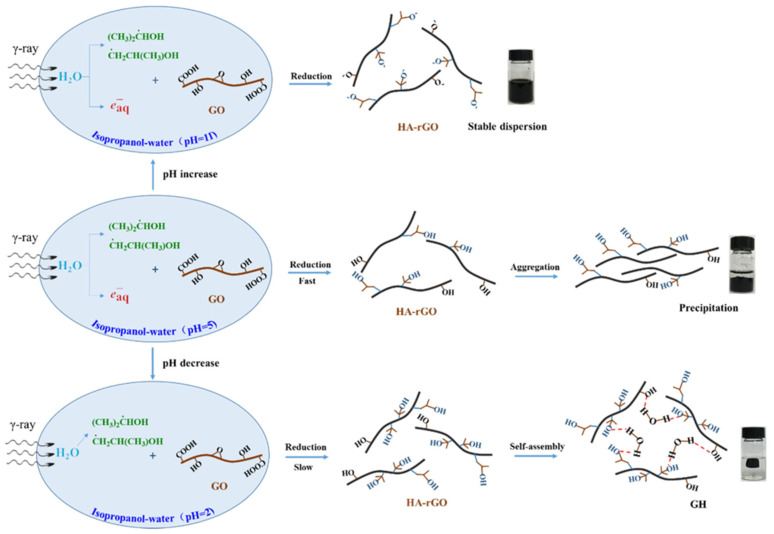
Effect of the pH of alcohol/water on GO reduction and the self assembly process. (Reproduced from Ref. [23] with permission).

Furthermore, the authors [23] of this research undertook an extensive investigation to explore how changes in the choice of alcohol/water solutions, the alcohol/water volume ratio (ϕa/w), and pH levels influence the reduction and self-assembly process of GO. Scheme 3, which illustrates the reaction mechanism using isopropanol as an example, provides valuable insights into these intricate processes. The key factor at play was the presence of highly reductive e_aq_^−^ species as the dominant active particles in the system (Figure 6).

In their comprehensive study, the researchers observed that the reduction degree of the product in the isopropanol/water dispersion with a pH of 5 was significantly higher than that in solutions with pH values of 2 or 11. In the pH = 5 environment, the reduction rate of GO nanosheets outpaced the binding rate of alcohol radicals, resulting in a notable increase in the hydrophobicity of hydroxyalkylated (HA)-rGO nanosheets. This, in turn, led to the formation of flocculent precipitates through π-π conjugation interactions.

As the pH level was raised to 11, both the alcohol radical concentration and the total number of reducing species decreased significantly, leading to a slower reduction rate of GO nanosheets. Conversely, when the pH was lowered to 2, all primary radicals were converted into alcohol radicals and the reactions were predominantly governed by hydroxyalkylation, resulting in the slowest reduction rate.

The study further uncovered that the reduction degree of GO nanosheets increased with higher absorbed doses and decreased initial GO concentrations. Moreover, a moderate ϕa/w ratio of 1/9.5 was found to be advantageous for the production of GH. The elongated linear hydroxyalkyl chains on the surface of GO nanosheets played a pivotal role in promoting the aggregation and stacking of these nanosheets, endowing the resulting graphene aerogel with excellent adsorption properties for various organic molecules. Notably, it exhibited a remarkable adsorption capacity for substances like chloroform and methanol, reaching 245 g/g and 158 g/g, respectively. These findings suggest that the prepared graphene aerogel has the potential to serve as a super-adsorbent for a wide range of compounds, including not only nonpolar oils but also polar alcohols, offering exciting prospects for diverse applications in environmental remediation and materials science.

### 2.2. Modified GA

**Figure 7:**
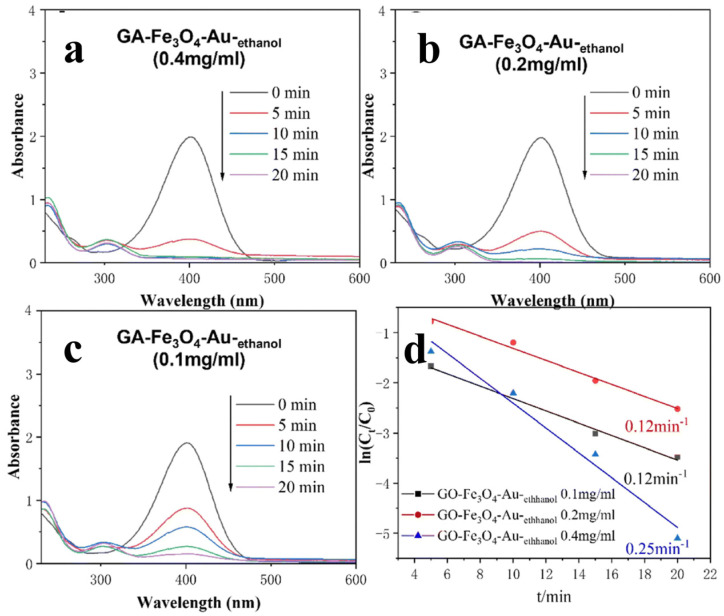
(**a**–**c**) UV−VIS spectrum of GA–Fe_3_O_4_–Au–ethanol for catalytic degradation of 4-NP. (**d**) The corresponding ln(C_t_/C_0_) versus time curve. (Reproduced from Ref. [26] with permission from the Royal Society of Chemistry).

To expand the application scope of GA, researchers have employed a novel approach involving γ-ray irradiation to reduce and deposit noble metal ions onto GA nanosheets. This innovative technique capitalizes on the unique properties of 3D porous GA, which provides an abundance of active sites for metal atoms, effectively preventing their agglomeration (Figure 7). This, in turn, enables the resulting composites to exhibit exceptional synergistic catalytic performance.

In a significant development back in 2016, Luo et al. [24] successfully introduced silver (Ag) ions into GO and crafted a 3D GA/Ag composite using a combination of γ-ray irradiation and freeze-drying in an isopropanol/water solution. This remarkable process not only led to the reduction in Ag ions and GO simultaneously but also ensured the uniform dispersion of Ag nanoparticles (NPs) on the surfaces of graphene sheets.

Building upon this foundation, in 2020, Lu et al. [25] took a step further by substituting the radical scavenger from isopropanol to absolute ethanol, which further enhanced the efficiency of the reduction process. Consequently, they obtained an ultralight self-supporting 3D GA/Ag composite through γ-ray irradiation-induced reduction in ethanol absolute. Moreover, the authors conducted a comprehensive evaluation of the catalytic performance of GA/Ag by employing the catalytic reduction in 4-nitrophenol (4-NP) to 4-aminophenol (4-AP) in the presence of the reducing agent sodium borohydride (NaBH_4_).

Thanks to the remarkable synergistic catalytic effect derived from the combination of graphene and Ag NPs, GA/Ag (with a mass ratio of GO to AgNO_3_ at 1:0.25) exhibited outstanding catalytic reduction activity for 4-NPs at both high (2.16 × 10^−3^ M) and low (0.72 × 10^−3^ M) concentrations. The first-order kinetic constants observed were 0.55 min^−1^ and 1.53 min^−1^, respectively, underscoring the exceptional catalytic potential of this composite. This advancement opens up new avenues for catalytic applications and further highlights the versatility and promise of graphene aerogels in various scientific and industrial domains.

**Figure 8:**
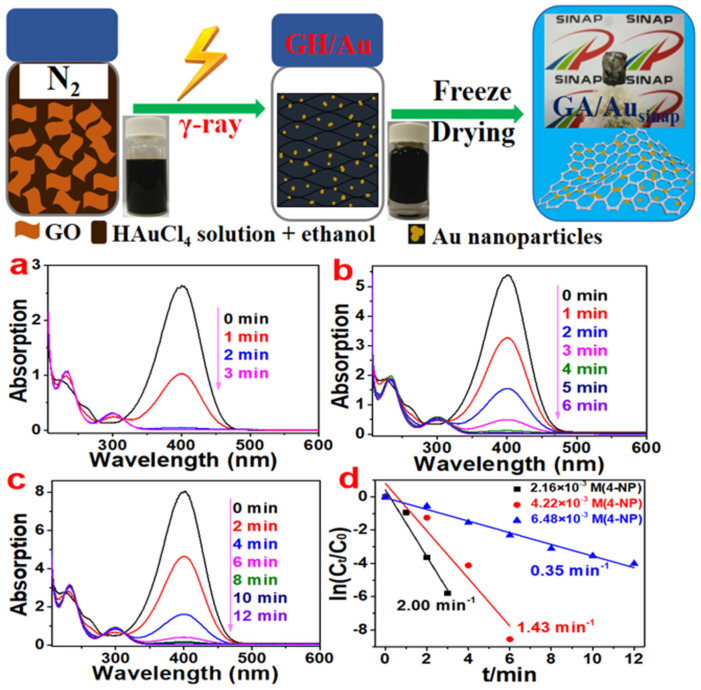
Preparation process of GA/Au composite materials. (**a**–**c**) Catalytic degradation of 4NP by GA/Au composite materials. (**d**) The corresponding ln(C_t_/C_0_) versus time curve. (Reproduced from Ref. [27] with permission).

Building on these successful techniques, the same research team (as mentioned in reference [27]) employed identical preparation methods to simultaneously reduce and self-assemble GO and gold ions (Au^3+^) in an ethanol/water solution. This innovative approach yielded a 3D graphene aerogel/gold (GA/Au) composite with an impressive high C/O ratio. The remarkable synergy in catalytic performance observed in the previous GA/Ag system was also evident in the case of GA/Au (Figure 8).

In the catalytic reduction of 4-NP, GA/Au demonstrated a similar level of outstanding performance. When the concentration of 4-NP was set at both 2.16 × 10^−3^ M and 4.22 × 10^−3^ M, the first-order kinetic constants were measured at 2.00 min^−1^ and 1.43 min^−1^, respectively. This underscores the effectiveness of the GA/Au composite in catalytic applications. Notably, it was observed that an optimal balance in the loading of Au NPs and the particle diameter is crucial, as either too little Au NP loading or excessively large particle diameters can affect the catalytic efficiency of the composite.

Furthermore, the researchers discovered an additional advantage of the GA/Au composite—it exhibited a notable adsorption capacity for organic solvents such as n-dodecane. This property suggests that GA/Au can effectively perform catalytic reactions in organic phases and can be applied in environmental remediation, broadening its potential applications and underscoring its versatility in addressing real-world challenges.

**Figure 9:**
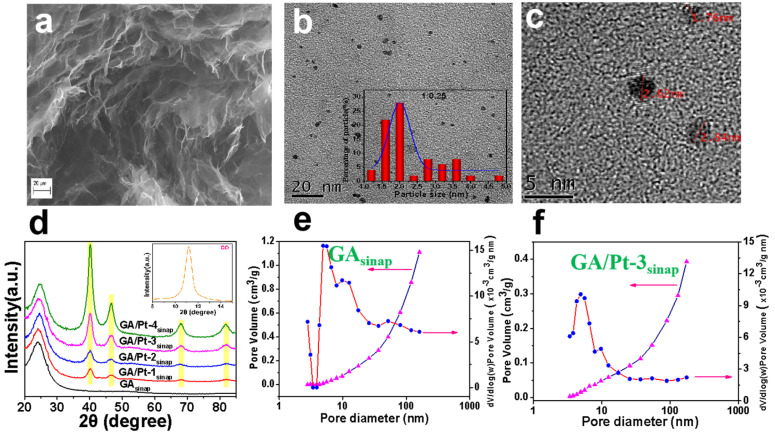
(**a**) SEM images of GA/Pt composite materials. (**b**) Uniformly distributed nano Pt particles on graphene sheets. (**c**) TEM images of GA/Pt composite materials. (**d**) Diffraction ring of GA/Pt composite materials. BET spectrums and the pore size distribution of (**e**) GA_sinap_ and (**f**) GA/Pt-3_sinap_. (Reproduced from Ref. [25] with permission).

In addition to the successful fabrication of Ag- and Au-containing graphene aerogels, researchers have also explored the deposition of Pt NPs onto graphene surfaces, opening up new possibilities for advanced materials. In 2020, Lu et al. [28] embarked on an exciting venture, utilizing ethanol as a radical scavenger to create GA/Pt composites through the unique combination of γ-ray irradiation and freeze-drying techniques (Figure 9).

This novel approach led to the uniform attachment of Pt NPs, resulting from the reduction in Pt^4+^, onto the graphene surface. The formation of hydrogen bonds further enhanced the interaction between Pt NPs and the graphene matrix. As the density of Pt NPs increased, so did the overall density of the GA/Pt composite. The graphene sheets not only expanded the active surface area of the composites but also mitigated the agglomeration of Pt NPs, fostering an accelerated electron transfer rate. As a consequence, the hydrogenation reduction reaction of GA/Pt towards methyl orange (MO) demonstrated remarkable performance.

The first-order kinetic constant for this catalytic reaction reached an impressive 1.773 min^−1^ when using a mass ratio of GO to H_2_PtCl_6_ at 1:0.25. Notably, this rate was 108 times higher than that of pure Pt NPs, highlighting the exceptional catalytic potential of the GA/Pt composite. Moreover, the authors discovered that the catalytic performance of GA/Pt is sensitive to the stirring speed. Intense stirring had the potential to break the composite material into a powder, significantly reducing the catalytic reduction time from 90 min with mild stirring to just 4 min, all without affecting the recovery rate.

Furthermore, Zhang et al. [29] contributed to this field by creating Pt/GA aerogels using γ-ray irradiation and freeze-drying but in an isopropanol/water solution. The Pt/GA-2 composite, with a GO to H_2_PtCl_6_·6H_2_O mass ratio of 1:2, exhibited mesoporous characteristics, evidenced by a type-IV sorption feature with a hysteresis loop in N_2_ adsorption–desorption. The Brunauer–Emmett–Teller (BET) specific surface area was exceptionally high, measuring 178.1 m^2^·g^−1^.

In a comparative assessment against several other samples and commercial Pt/C, Pt/GA-2 outperformed the rest by displaying the fastest catalytic reduction rate constant, clocking in at 1.14 min^−1^·mg^−1^, and the lowest activation energy value, a mere 23.16 kJ·mol^−1^. These findings not only underscore the versatility of graphene aerogels but also highlight the remarkable potential of Pt/GA composites for various catalytic applications, paving the way for more environmentally friendly and efficient catalytic processes.

In addition to the incorporation of noble metal nanoparticles, researchers have explored the integration of metal oxides into GA, further diversifying the range of advanced composite materials. A notable contribution in this direction comes from Fan et al. [30] who devised a method to introduce tin dioxide (SnO_2_) nanoparticles into graphene aerogels.

Their innovative approach involved the oxidation of Sn^2+^ ions to SnO_2_ nanoparticles through the application of γ-ray irradiation in an ethanol/water solution. The resulting SnO_2_ nanoparticles displayed an intriguing affinity for interaction with rGO sheets within the GA@SnO_2_ composite. These interactions encompassed physical adsorption, electrostatic binding, and charge transfer phenomena, collectively fostering a conducive environment for the reduction process of GO.

This research not only showcases the versatility of graphene aerogels as a platform for integrating metal oxides but also underscores the potential of such composites to facilitate and enhance various chemical processes, opening up new avenues for applications in materials science and catalysis.

**Scheme 4:**
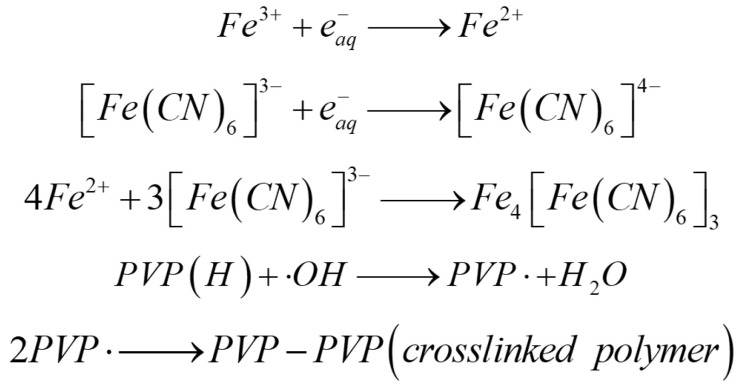
Reactions mechanism of PB@PVP/rGO aerogel.

**Figure 10:**
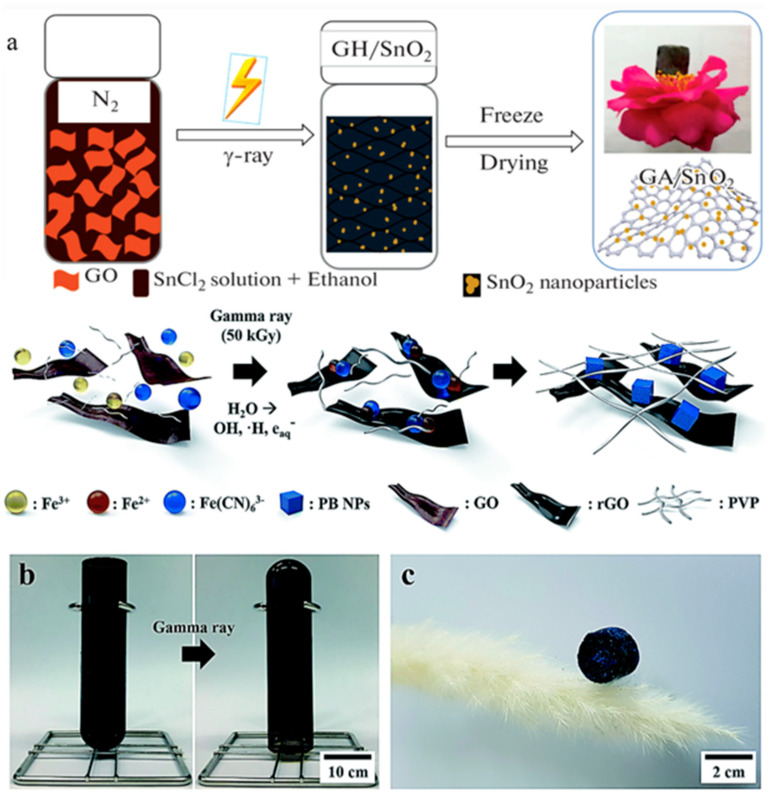
(**a**) Preparation of GA/SnO_2_ composite materials. (**b**,**c**) Preparation of Prussian blue (PB)@PVP/rGO aerogel. (Reproduced from Refs. [30,31] with permission).

There are also research works to prepare GA without additional radical scavengers. In 2019, Lee et al. [31] prepared Prussian blue (PB)@PVP/rGO aerogel by reducing PVP, K_3_Fe(CN)_6_, FeCl_3_, and GO solutions via γ-ray irradiation, Scheme 4. Because the PB nanoparticles were introduced into the double network structure composed of PVP and rGO, the hydrophilicity of the aerogel was improved and the aerogel had a rapid adsorption effect on Cs^+^ ions and MB dyes under the capillary action (Figure 10). According to the Langmuir model, the maximum adsorption amounts of Cs^+^ and MB were 143.88 and 44.73 mg·g^−1^, respectively. In addition, the swelling ability of the aerogel was affected by pH and its swelling rate increased when the pH value was lower.

## 3. Conclusions

Radiation-based preparation of GA typically involves utilizing GO as the precursor material. The reduction and self-assembly of GO are induced by γ-ray irradiation to yield a graphene hydrogel. Subsequently, a freeze-drying method is employed to obtain a lightweight GA characterized by a high carbon-to-oxygen (C/O) ratio. It is crucial to note that various factors can influence the reduction and self-assembly process during this approach, including the absorbed dose, solution composition, pH value, and concentration.

To enhance the versatility and application potential of GA, additional techniques can be incorporated. Amines or alcohols are often introduced as free radical scavengers to capture and neutralize species like e_aq_^−^, ·OH, and other particles generated through water radiolysis. This not only helps in eliminating oxygen-containing groups but also facilitates the introduction of nitrogen-containing groups or hydroxyl alkylation. Moreover, in a bid to expand the scope of GA applications, metal ions can be introduced into the mixed solution. This has the dual benefit of simultaneously reducing the metal ions and GO while ensuring even dispersion of nanoparticles on the graphene sheets.

The graphene aerogels produced through irradiation methods offer numerous advantages and exhibit substantial potential in applications such as organic solvent absorption, oil spill cleanup, and environmental remediation efforts. However, it is worth mentioning that, despite these promising attributes, there remains a scarcity of research on the radiation-based preparation of graphene aerogels.

In summarizing the aforementioned content, the radiation-based preparation of GA reveals significant prospects for both research and practical applications. A deeper understanding of the process points towards several key directions for future developments. Firstly, a more in-depth optimization of process parameters will be a crucial focus. Adjusting factors such as absorbed dose, solution composition, and pH value can enhance the quality and performance of graphene aerogels, enabling precise control over their structure. Secondly, improving the multifunctionality of graphene aerogels is a challenging yet promising avenue. Introducing new functional materials or enhancing free radical scavengers to broaden their applicability across different domains will enhance the practicality of this technology. Expanding the application areas is another pivotal direction for the future. In-depth exploration of graphene aerogels in real-world applications such as organic solvent absorption, oil spill cleanup, and environmental remediation will uncover new scenarios, driving the technology’s application in solving practical problems. Additionally, considering the introduction of different metal ions or materials to synthesize innovative graphene aerogels holds potential for improving their performance and applicability. This opens up new possibilities in the field of materials science. Lastly, conducting more systematic and comprehensive studies to understand the impact of radiation-based preparation methods on the structure and performance of graphene aerogels will help address current knowledge gaps and propel the field towards deeper breakthroughs.

Through continuous research and innovation, there is a promising outlook for fully unlocking the potential of radiation-based graphene aerogel technology, offering new possibilities in both scientific research and practical applications. Therefore, further research and development in this innovative technology are deemed crucial.

## Figures and Tables

**Table 1:** Comparison of the main features of various 3D graphene-based aerogels.

Architectures	Adsorbed Dose [kGy]	Radical Scavenger	C/O Radio	Pore Size [µm]	NPs Size (1)	Mass Density [mg cm^−3^]
RGO aerogel ^19,20^	400	Ethylenediamine	9.56	8–15	None	3.5–4.0
GA ^21^	200	Isopropanol	12.0	10	None	3.82
GA ^22^	>250	t-butanol	5.6	—	None	4
GA ^23^	54.7	Isopropanol	13.0	—	None	4.0
GA/Ag ^24^	200	Isopropanol	—	2–15	20–80	—
GA/Ag ^25^	300	Ethanol	—	—	20–60	3.2–5.7
GA/Au ^26^	—	Ethanol	14.13	—	6–28	4.5–5.6
GA/Pt ^27^	—	Ethanol	8.8	2–10	2–10	3.4–5.9
Pt/GA ^28^	200	Isopropanol	Decreased significantly	—	3	—
GA@SnO_2_ ^29^	—	Ethanol	—	10	3–5	10.82
PB@PVP/rGO aerogel ^30^	50	No	—	38.66 ± 4.20 98.28 ± 7.77	57.94 ± 3.10	3.1

## Data Availability

Not applicable.
